# Compression of frailty in adults living with HIV

**DOI:** 10.1186/s12877-019-1247-3

**Published:** 2019-08-22

**Authors:** Giovanni Guaraldi, Davide De Francesco, Andrea Malagoli, Stefano Zona, Iacopo Franconi, Antonella Santoro, Cristina Mussini, Chiara Mussi, Matteo Cesari, Olga Theou, Kenneth Rockwood

**Affiliations:** 1 Department of Surgical, Medical, Dental and Morphological Sciences, University of Modena and Reggio Emilia, Largo del Pozzo, 71, 41124 Modena, Italy; 2 Institute for Global Health, UCL, London, UK; 3 Fondazione IRCCS Ca’Granda, Ospedale Maggiore Policlinico, Milan, Italy; 4 Geriatric Medicine Research Unit, Department of Medicine, Dalhousie University & Nova Scotia Health Authority, Halifax, Nova Scotia Canada

## Abstract

**Background:**

Contemporary HIV care may reduce frailty in older adults living with HIV (OALWH). Objective of the study was to estimate prevalence of frailty at the age of 50 and 75 years, and build a model to quantify the burden of frailty in the year 2030.

**Methods:**

This study included OALWH attending Modena HIV Metabolic Clinic between 2009 and 2015. Patients are referred from more than 120 HIV clinics well distributed across Italy, therefore being country representative. Our model forecasts the new entries on yearly basis up to 2030. Changes in frailty over a one-year period using a 37-variable frailty index (FI) and death rates were modelled using a validated mathematical algorithm with parameters adjusted to best represent the changes observed at the clinic. In this study, we assessed the number of frailest individuals (defined with a FI > 0.4) at the age of 50 and at the age 75 by calendar year.

**Results:**

In the period 2015–2030 we model that frailest OALWH at age 50 will decrease from 26 to 7%, and at the age of 75 years will increase from 43 to 52%. This implies a shift of the frailty prevalence at an older age.

**Conclusion:**

We have presented projections of how the burden of frailty in older adults, living with HIV will change. We project fewer people aged 50+ with severe frailty, most of whom will be older than now. These results suggest a compression of age-related frailty.

**Electronic supplementary material:**

The online version of this article (10.1186/s12877-019-1247-3) contains supplementary material, which is available to authorized users.

## Background

The World Health Organization (WHO) views population aging as one of the most important demographic changes in society [1]. Globally, the proportion of elderly people is growing at 2% per year, much faster than the population as a whole [1]. Worldwide, between 2000 and 2050, the proportion of the people over 60 years is expected to double.

Aging of the population is becoming a top priority in health policy [2]. With aging comes an increased prevalence of most diseases, which tend to co-occur in older people [2]. In this context, the demographic modifications occurring among HIV patients are not an exception but rather a paradigm of this changing scenario [3]. Globally 35.3 million people are living with HIV, and among them an estimated 3.6 million are people aged 50 years or older [4]. Older adults living with HIV (OALWH) represent a heterogeneous population almost equally distributed between people aging with HIV and people who acquired HIV at an older age [5]. The advent of early, effective combination antiretroviral therapy (ART) and contemporary HIV care has allowed an increasing number of HIV patients to live into old age. The median age of HIV-positive patients receiving ART is projected to increase from 43.9 in 2015 to 56.6 in 2030, as the proportion of HIV-positive patients aged 50+ years increases from 28 to 73% during the same timeframe. With aging, the clinical complexity in the HIV-positive population will increase. For example, the number of people with one non-communicable disease (NCD) is expected to be 84% by 2030, and the proportion with multi-morbidity (3+ illnesses) to be 28% [6].

Geriatric medicine, more than twenty years ago, has introduced the term “frailty” to define a condition caused by the reduction of homeostatic reserves exposing the individual to higher risk of negative outcomes [7].

Frailty is an independent predictor of several adverse clinical outcomes in older persons, including multi-morbidity (MM), falls, disability, nursing home placement, and death [8]. In particular, the detection of frailty has been repeatedly advocated for taking preventive actions against disability in older persons [9, 10].

Both in the general populations and in HIV/AIDS setting, frailty has been used to define a specific geriatric syndrome [11] but also as a state of vulnerability for adverse outcomes [12]. Frailty is considered an interval parameter reflecting the “biological age” of the individual, and might represent much more than a mere condition to be screened. As argued recently, it may indeed meet the criteria for making public health decisions [10]. In this framework frailty allows to assess clinical and biological features, which have been indecently shown to robustly affect the vulnerability of individuals, notably in their mortality risk, regardless chronological age, [13]. Several reports have described that the prevalence of frailty is double in HIV patients when compared to HIV negative matched controls [14]. In this context, similarly to what public health is pursuing in compressing chronic diseases to a shorter period later in life [15, 16], we hypothesized that contemporary integrated care in HIV medicine may progressively lead to a “compression of frailty at an older age” with reduction of frailty in adults living with HIV and restrain frailty to a later stage of age. This study estimates the prevalence of frailty in OALWH at the age of 50 and 75 years, and presents a model estimating the burden of frailty up to 2030.

## Methods

The Modena HIV Metabolic Clinic (MHMC) is a tertiary level center for diagnosis and treatment of lipodystrophy, NCDs and frailty in HIV people, started in 2003. HIV patients attending MHMC are referred from more than 120 HIV clinics well distributed across Italy therefor being country representative. Previous sensitivity analyses showed that referred patients are similar to local (Modena province) whole HIV cohort with no case selection bias [15].

The service prospectively follows patients with free-of-charge visits with infectious disease physicians, geriatricians, occupational therapists, nutritionists, psychologists, cardiologist, nephrologists, endocrinologists, and plastic surgeon consultants. At each visit, patients receive a multidimensional evaluation, including clinical, anthropometric, physical performance, and neurocognitive assessments. All the data are recorded in a dedicated electronic health record which integrates health care workers’ assessments with patients related outcomes, including geriatric syndromes (falls, urinary incontinence, etc.), quality of life and disability. The MHMC electronic health record provides a standardized comprehensive tool to describe changes of frailty over time at an individual level. The present analyses include 2982 ART-treated HIV-positive subjects, aged more than 18 years, who were consecutively evaluated at the Metabolic Clinic of the University of Modena and Reggio Emilia between 2006 and 2015. Demographic factors of interest included age and sex. HIV-specific characteristics included HIV and antiretroviral therapy (ART) duration, nadir and current CD4^+^ T cell count.

### Frailty index (FI)

Frailty was operationalized using a Frailty Index (FI) which was previously validated and included 37 health variables routinely collected at each clinical visit (Additional file 1: Table S1), [17]. Each variable included in the FI was coded with the value of 1 when a deficit was present and 0 when absent. The FI for each patient visit was calculated as the ratio between the number of deficits present and the total number of deficits assessed. Missing values were removed from both the numerator and denominator of the FI. Each FI was computed when a minimum of 80% of valid data for the health variables was available [18]. Of note, the health variables that were selected as outcomes or covariates of interest for this study were excluded from the computation of the FI.

The FI has been found to predict risk in increments as low as 0.01, as well as 0.10. For a clinical study, we chose increments of 0.10. Recognizing that there would be very few people with low FI counts in a clinical sample, and that there is poor survival at high FI scores we grouped participants into the following four categories: fit < 0.2, pre frail 0.2–0.3, frail 0.31–0.39, and frailest > 0.40 [18].

In this study a novel metric, termed the frailty compression ratio (FCR), was developed to determine the change in relation between frailty and chronologic age over time. The FCR was defined as the fraction between the number of frailest individuals (defined as those with an FI > 0.4) at the age of 75 divided by the number of frailest individuals at the age of 50 among OALWH attending the MMHC. An increase in this ratio represents a shift of the prevalence of frailty towards an older age.

### Multimorbidity

Multi-morbidity was defined as ≥3 NCDs occurring in the same individual among hypertension, diabetes, cardiovascular disease (CVD), dyslipidaemia, chronic obstructive pulmonary disease (COPD), chronic kidney disease (CKD), fractures, liver cirrhosis and cancer [19].

### Ethics

Approval for the Modena HIV Metabolic Clinic Cohort was obtained from the Research Ethics Board of the University of Modena and Reggio Emilia, and all participants provided written consent (Study reference 254/12, protocol 883/CE, date 7th March 2013).

### Statistical analysis

Using clinic data collected between 2006 and 2015, we constructed an individual-based model of the aging population attending the MHMC. The model follows patients enrolled in the MHMC up to December 2015, until death or end of the model simulation in 2030, estimating outcomes such as FI and MM at one-year time intervals. Similarly, the model generates new entries into the MHMC for each year from 2016 to 2030 and follows them (until death or end of 2030) on a yearly basis.

Predictions of the model for the first two years (2016 and 2017) were validated using data collected in the corresponding years to ensure projections were pointing in the right direction.

The number of new entries from 2016 to 2030 was simulated projecting trends observed from 2006 and 2015 in the MHMC. Gender was assigned probabilistically to new patients using the observed male to female ratio. Age of each new patient was randomly generated from a gamma distribution with gender- and year-specific parameters (mean and standard deviation) projected from trends observed between 2006 and 2015. Similarly, nadir CD4^+^ T cell count and time since HIV infection were randomly generated from normal distributions with age-, gender- and year-specific parameters.

The prevalence of each NCD was determined in the whole population and in age-defined groups (18–39, 40–44, 45–49, 50–54, 55–59, 60–64 and ≥ 65) and Chi-squared test for trend was used to compare the prevalence of NCDs across age groups and test for significant trends. Continuous outcomes were summarised using the median and interquartile range (IQR).

Occurrence of MM at model entry was probabilistically assigned to new patients based on rates modelled using age, gender, time since HIV infection and nadir CD4^+^ T cell count. The contribution of each of these factors to estimate the rate was obtained from a multivariable mixed-effect logistic regression model. For each simulated year, new patients and patients already enrolled in the MHMC before 2016 were followed as they age and eventually die, simulating changes in FI and MM.

The number of deficits (used to calculate the FI) at the time of entry into the MHMC for each new patient was randomly generated from a Poisson distribution with mean depending on age, gender, time since HIV infection, and nadir CD4^+^ T cell count. Weights for each of these factors (to estimate the mean of the Poisson distribution) were obtained from a multivariable mixed-effect Poisson regression model predicting the FI in the population of the MHMC from 2006 to 2015. Changes in FI over a year were modelled using a normal distribution with parameters depending on age, gender, time since HIV infection, nadir CD4^+^ T cell count and FI in the previous year. The generated number was then added to the FI of the previous year.

Death rates were obtained from a validated mathematical model which estimates the probability of dying in a given period of time based on the FI at the beginning of the period of time. This model was developed in a large Canadian aging population [20] and parameters were adjusted to best represent the death rates observed in the MHMC.

The probability of having a FI score > 0.40 (frailest individuals) and that of MM at any given year was estimated using polynomial logistic regression models with age as independent variable and with the degree of the polynomial that minimized the variance of the model or when there was no significant decrease in its value as the degree of polynomial is increased. The probability of being at the frailest category (i.e. FI > 0.40) for individuals of 50 and 75 years was then calculated at each year between 2016 and 2013 and used to estimate the frailty compression ratio (FCR) for coming years.

Linear regression models were used to test significant time trends (from 2006 to 2015) in the distribution of age, time since HIV infection and start of ART, nadir CD4+ T cell count, proportion of late presenters and patients referred to the clinic after 20 years or more since HIV infection. Models were conducted separately in women and men and were aimed at identifying changing factors in MHMC in the period 2009–2015 which may have justified model’s predictions.

All the analyses (including model simulations) were performed using the statistical software R version 3.3.1.

## Results

The 2982 HIV-positive patients enrolled in the MHMC and alive at the end of December 2015 were mainly men who were taking anti-retroviral therapy (Table 1).

**Table 1:** Socio-demographic, anthropometric lifestyle and HIV-specific characteristics

Variable, n (%) or median (IQR)	HIV-positive (*N* = 2982)
Gender	
Female	951 (31.9%)
Male	2031 (68.1%)
Age (years)	49 (45, 54)
Weight (kg)	68.0 (59.6, 76.6)
Height (cm)	170 (163, 176)
BMI (kg/m^2^)	23.5 (21.4, 26.0)
Waist (cm)	87 (81, 94)
Hip (cm)	92 (88, 97)
Waist-to-Hip ratio	0.95 (0.90, 1.00)
Smoking	
No smoking	1756 (58.9%)
1–10 cigarettes per day	516 (17.3%)
> 10 cigarettes per day	672 (22.5%)
*Missing*	38 (1.3%)
Alcohol consumption	
No consumption	1766 (59.2%)
Mild consumption	1149 (38.5%)
Intense consumption	31 (1.1%)
*Missing*	36 (1.2%)
Physical activity	
No physical activity	1484 (49.8%)
Mild physical activity	1206 (40.4%)
Intense physical activity	265 (8.9%)
*Missing*	27 (0.9%)
Likely route of transmission, n (%)	
Homosexual sex	881 (29.5%)
Heterosexual sex	1014 (34.0%)
IVDU/Blood product	778 (26.1%)
Other/unknown	309 (10.4%)
CDC classification, n (%)	
A	1248 (41.9%)
B	862 (28.9%)
C	699 (23.4%)
*Missing*	173 (5.8%)
Years since HIV diagnosis	19.7 (12.8, 24.4)
Currently on ART	2810 (92.5%)
Time since start of ART (years)	5.2 (2.6, 7.8)
Number of drugs	3 (3, 4)
Currently on NRTIs	2319 (82.5%)
Currently on PIs	1550 (55.2%)
Currently on NNRTIs	1103 (39.3%)
Currently on other drugs	569 (20.6%)
CD4^+^ T cell count (cells/μL)	648 (474, 841)
CD8^+^ T cell count (cells/μL)	815 (601, 1113)
CD4^+^: CD8^+^ ratio	0.80 (0.57, 1.10)
CD4^+^ T cell count (cells/μL)	200 (86, 300)

Socio-demographic, anthropometric, lifestyle and HIV-specific characteristics are summarized in Table 1. Median time since HIV diagnosis was 19.7 (IQR 12.8, 24.4) years and the median nadir CD4+ T cell count was 200 (IQR 86, 300) cells/μL.

Among the NCDs, most participants (82.1%) had dyslipidemia, followed by hypertension (36.8%) osteoporosis (20.5%) and cirrhosis of the liver (12.3%). Fewer than 5% had type 2 diabetes, CVDs, cancer, COPD and CKD. The prevalence of all NCDs increased significantly with age (Additional file 1: Figure S1).

**Fig. 1:**
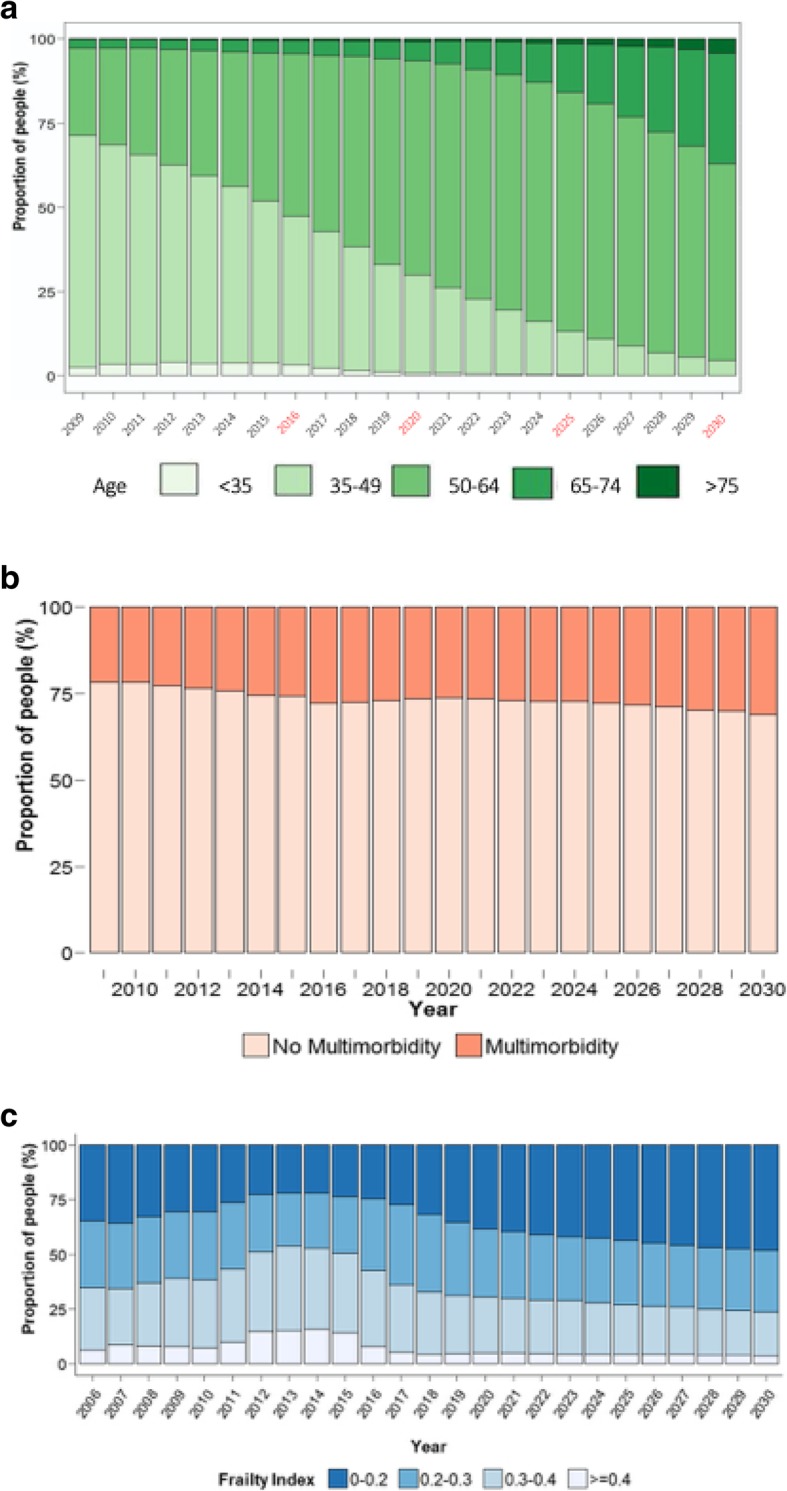
Prediction of median age, multi-morbidity and frailty in PLWH. Panel **a**. The median age predictions of patients from 2015 to 2030. Panel **b**. The proportion of patients with multi-morbidity from 2015 to 2030. Panel **c**. The distribution of frailty index values from 2015 to 2030

The model predicts that the median age of patients evaluated at MHMC will increase from 49 (IQR 45, 54) years in 2015 to 63 (IQR 58, 77) years in 2030. The proportion of patients older than 50 years is predicted to increase from 48.2% in 2015 to 95.6% in 2030, whereas the proportion of patients aged 65 years or older will increase from 4.3 to 38.1%. In particular, the proportion of people aged 75 years or older will increase from 0.6 to 4.3% (Fig. 1, panel A). As a result of the aging HIV-positive population, the number of patients with MM is expected to increase from 52.3% in 2015 to 62.1% in 2030 (Fig. 1, panel B). Our model suggests that the proportion of frail patients (FI > 0.3) will increase from 25.8% in 2015 to 28.0% in 2030 and frailest patients (FI > 0.4) will increase from 23.8 to 48.2% (Fig. 1, panel C).

**Fig. 2:**
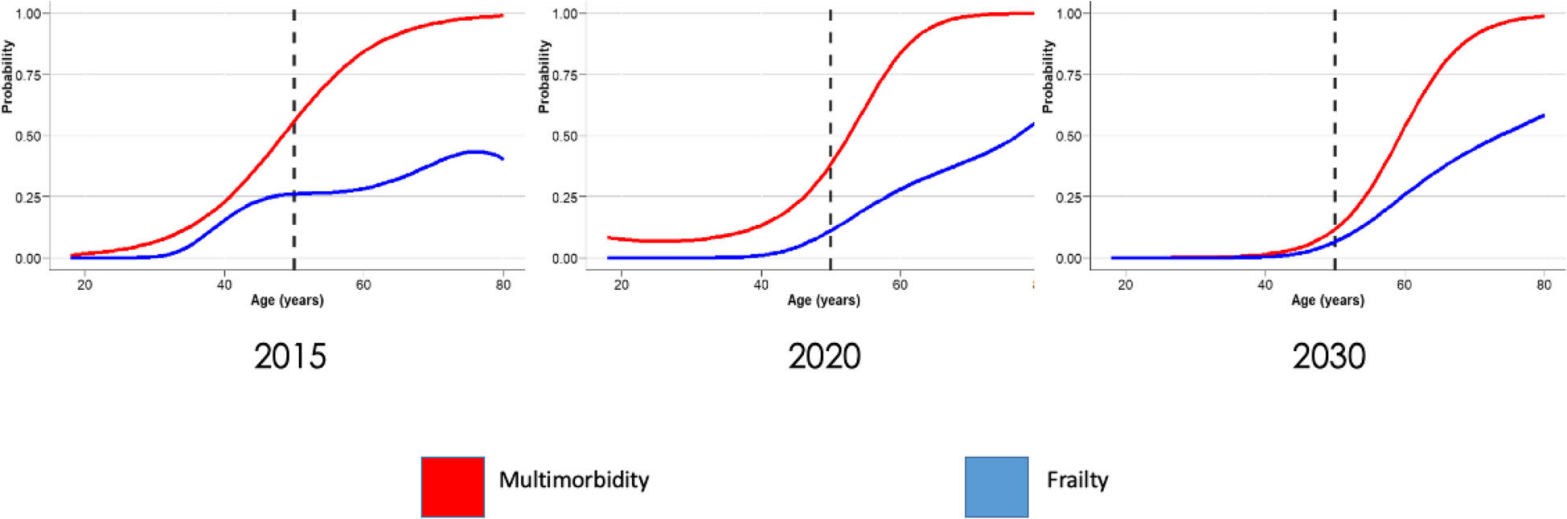
The probability of multi-morbidity and frailty in 2015, 2020 and 2030

Figure 2 estimate that the probability of MM at age of 50 decreased from 37.8 to 11.7% while at the age of 75 the probability of MM went from 99.8 to 96.8%. In parallel, there was a significant reduction of the probability of frailty at the age of 50 from 26.0 to 7.0%, and an increase at the age of 75 from 42.7 to 51.8% in the same time frame.

**Fig. 3:**
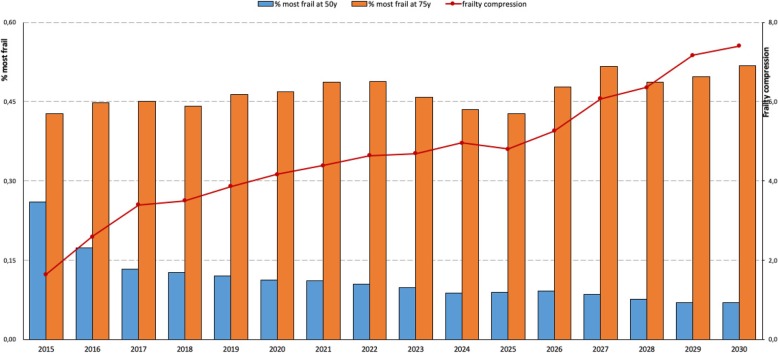
Projections of the proportion of frailest individuals at the age of 50 (blue bar) and at the age of 75 (orange bar)

Figure 3 shows projections of the proportion of frailest individuals at the age of 50 (blue bar) and at the age of 75 (orange bar). The ratio of these two proportions at each year is the FCR and it is represented by the red dotted line. FCR will increase from 1.6 to 7.4 from 2015 to 2030.

We explored the factors that may have contributed to the FCR. Proportion of male patients (rho = 0.71, *p* < 0.01), age (rho = 0.56, *p* = 0.056), nadir CD4+ T cell count (rho = 0.86, *p* < 0.001) and proportion of patients with HIV duration more than 20 years (rho = 0.84, *p* < 0.001) correlated with frailty compression in univariable analysis. Association was not statically significant for time exposure of antiretroviral treatment (ART), time lag between HIV diagnosis and ART initiation and proportion of HIV late presentation (CD4 < 350 cells/μL).

Proportion of male patients (*p* = 0.02), age (*p* < 0.001), nadir CD4+ T cell count (p < 0.001) and proportion of patients with HIV duration more than 20 years (*p* = 0.001) remained significantly associated even in multivariable analysis.

## Discussion

This is the first modelling study quantifying the burden of MM and frailty in relation to demographic projections estimated in an HIV Italian cohort. This model shows an increase in the burden of MM and frailty in the year 2030, indicative of an accentuated health profile expected by the aging epidemic affecting OALWH, as previously shown by the Athena cohort projections [4].

Frailty as primary end point of this study reflects a geriatric approach which considers frailty as the best indicator to describe the complexity and heterogeneity of aging.

We chose to operationalize frailty with FI rather than with frailty phenotype because given the continuous metric of former, make this tool more suitable to describe trajectories over time [21].

Being based on arithmetic and not clinical assumptions, frailty index is more suitable than others for an immediate translation of the frailty concept from geriatrics to the HIV medicine world given its quantitative nature [22].

The burden of frailty in OALWH by the year 2030 should not be interpreted as an apocalyptic scenario since this projection shows that these conditions will be compounded to older age. In fact, it is not alarming that 50% of HIV patients will be most frail at the age of 75, it simply shows that the aging HIV-positive population may gradually get closer to the clinical manifestations of the general geriatric population.

It may be questioned the reason why at present, a relatively low number of people aged > 75 are frail. First of all, people belonging to this age group are few. Moreover, it can also be assumed a survival bias and therefore people in this age group may really represents “champions” of their generation, being able to live longer with HIV infection or getting HIV at an older age [17].

The crux of the study is the development of the concept of frailty compression at an older age. To our knowledge this measure, that we have translated into an algorithm which consider the ratio of the proportion of frailest individuals at the age of 75 and at the age of 50 years at any given year, is original in geriatric medicine also. Given the tight connection between MM and frailty, we believe that measuring the deficit accumulation will allow us to monitor trajectories of a healthy aging, regardless of the co-occurrence of age-associated NCDs.

The striking finding from our cohort is that the prevalence of frailest individuals at the age of 50 years decreased in the past decade. What happened in OALWH attending MHMC is a unique and favorable phenomenon of frailty compression to older age. This was associated with a profound changing in immunological characteristics of the patients depicted by higher nadir CD4 and a higher proportion of people living with HIV longer than 20 years. The former may document a reduction in the proportion of OALWH with a permanent immunological scar associated with advanced HIV disease, the latter, healthier patients living with HIV with better immunological profile.

We are not aware of other disease model in which frailty compression was so apparent: at MHMC the proportion of frailest individual at 50 years of age halved in the past 10 years. Therefore, we hypothesize that the immunological characteristics which stand behind this favorable immunological improvement observed in OALWH may be found also in HIV negative individuals experiencing healthy aging.

Recognition of frailty change in term of prevalence and age of onset in HIV patients will contribute to reshape our healthcare systems. HIV geriatric medicine implies a structural and a cultural change in patient assessment focused on older person’s functional ability, physical health, cognition, quality of life and socio-environmental circumstances rather than assessing patient’s comorbidities [22].

Interdisciplinary team members (e.g. nurses, social workers, pharmacists, psychologists and occupational therapists) can administer screening tools to both save time and help the team to focus on specific limitations that need more detailed evaluation. The goal of prevention is there for not just avoiding mortality as an obvious negative end point, but rather reaching a disability free survival in a healthy aging process [3].

Some weakness of the study must be acknowledged. The projection model is obviously itself a model therefore prone to errors. Second, it is based on the retrospective analyses of patients enrolled in a clinical cohort which may not generalize to the whole HIV-positive population worldwide.

A point of strength of the study is the original use of FI as an epidemiological tool to make a projection using a global health perspective. The unprecedented rapid change of clinical care observed in the past 30 years, once again pilot new epidemiological scenarios for public health in which the comprehensive geriatric assessment evaluates frailty at screening and contemporary targets this condition in an adapted individualized approach [23]. This approach particularly fits PLWH where many individuals are frail regardless their relatively young age [24] and allows a holistic and personalized care.

## Conclusions

In conclusion, we have presented projections of how the burden of frailty in older adults, living with HIV will change. We project fewer people aged 50+ with severe frailty, most of whom will be older than now. These results suggest a compression of age-related frailty.

## Additional file

Additional file 1: **Figure S1.** Prevalence of comorbidities by age group. **Table S1.** Health variables included in the frailty indices and description of deficit scoring. (PDF 414 kb)

## Data Availability

The datasets used and/or analysed during the current study are available from the corresponding author on reasonable request.
