# Supplementary material for: Compression of frailty in adults living with HIV
Source: BMC Geriatr. 2019 Aug 22;19:229. doi: 10.1186/s12877-019-1247-3 (PMC6706922; doi:10.1186/s12877-019-1247-3)
Supplement: Supplementary file 1 — Figure S1. Prevalence of comorbidities by age group. Table S1. Health variables included in the frailty indices and description of deficit scoring. (PDF 414 kb) [file 12877_2019_1247_MOESM1_ESM.pdf]

Suplementary material Figure 1.
Prevalence of comorbidities by age group

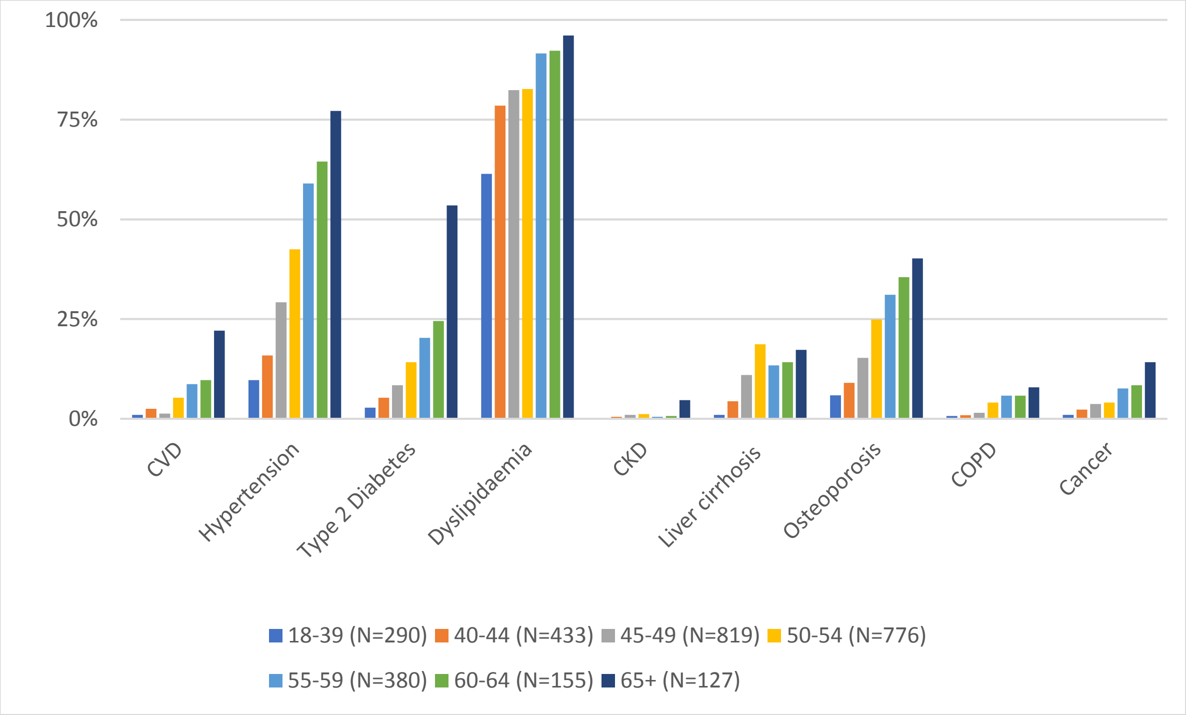

Supplementary Table 1.

Health variables included in the frailty indices and description of deficit scoring

No.	Variable	Deficit description
	**37-item frailty index**	
1	Lipoatrophy	• Multicenter AIDS Cohort Study (MACS) criteria (50)
2	Lipohypertrophy	• MACS criteria (43)
3	Non-alcoholic fatty liver disease	• Liver/spleen ratio < 1.1
4	Menopause or male hypogonadism	• If female: FSH>30 IU/L & LH<30 IU/L and/or absence of menstruation >1 year<br>• If male: testosterone<300 ng/dL
5	High or low body mass index	• <18 or >25 kg/m^2
6	High waist circumference	• If female: >88 cm<br>• If male: >102 cm
7	High visceral adipose tissue	• VAT>130 cm^2 or VAT/TAT ratio >0.5
8	Sarcopenia or presarcopenia	• Fat-free mass index < -1 SD
9	Insulin resistance	• Homeostasis Model Assessment – Insulin Resistance (HOMA-IR) (51) > 2.8
10	High total cholesterol	• > 200 mg/dL
11	High low density lipoprotein	• >100 mg/dL
12	Low high density lipoprotein	• < 40 mg/dL
13	High triglycerides	• >150 mg/dL
14	High homocysteine	• If female, > 10 µmol/L<br>• If male, > 15 µmol/L
15	Abnormal white blood cell counts	• < 4000 cells/µL
16	Anemia	• If female, < 10 grams/dL<br>• If male, < 12 grams/dL.
17	Hepatitis C co-infection	• Positive
18	Hepatitis B co-infection	• Hepatitis B Antigen positive

19	Vitamin D insufficiency	• < 30 ng/mL
20	Polypharmacy	• > 5 drug classes (excluding antiretroviral therapy)
21	Abnormal parathyroid hormone	• > 60 pg/mL
22	Elevated D-dimer	• > sample mean (358)
23	Elevated C-reactive protein	• > 0.7 mg/L
24	Sedentary lifestyle	• < 3 hours/week physical activity
25	Atherosclerosis	• Coronary artery calcium score > 100 or intima media thickness > 0.85 mm
26	Hyponatremia	• < 125 mmol/L
27	Proteinuria or albuminuria	• > 5 mg/mmol
28	Elevated aspartate transaminase (AST)	• > 31 U/L
29	Elevated alanine transaminase (ALT)	• > 31 U/L
30	Abnormal alkaline phosphatase	• < 38 or > 126 U/L
31	Elevated gamma-glutamyl transphosphatase (GGT)	• > 55 U/L
32	Low platelets	• < 150 billion/L
33	Abnormal potassium	• < 3·5 or > 5·3 mEq/L
34	Abnormal phosphorus	• < 2·5 or > 5·1 mg/dL
35	Abnormal thyroid stimulating hormone	• < 0·27 or > 4·2 mIU/L
36	Elevated total bilirubin	• > 1·10 mg/dL
37	Unemployment	• Self-report
